# Distinct gut and vaginal microbiota profile in women with recurrent implantation failure and unexplained infertility

**DOI:** 10.1186/s12905-022-01681-6

**Published:** 2022-04-12

**Authors:** Nayna Patel, Nidhi Patel, Sejal Pal, Neelam Nathani, Ramesh Pandit, Molina Patel, Niket Patel, Chaitanya Joshi, Bhavin Parekh

**Affiliations:** 1 Akanksha Hospital and Research Institute, Lambhvel Road, Lambhvel, Anand, Gujarat 387310 India; 2 Gujarat Biotechnology Research Centre (GBRC), Gandhinagar, Gujarat 382011 India; 3 Present Address: School of Applied Sciences & Technology (SAST-GTU), Gujarat Technological University, Visat - Gandhinagar Road, Chandkheda, Ahmedabad, Gujarat 382424 India

**Keywords:** Gut microbiota, Vaginal microbiota, Implantation failure, Infertility, Dysbiosis

## Abstract

**Background:**

Female reproductive tract dysbiosis impacts implantation. However, whether gut dysbiosis influences implantation failure and whether it accompanies reproductive tract dysbiosis remains scantly explored. Herein, we examined the gut-vaginal microbiota axis in infertile women.

**Methods:**

We recruited 11 fertile women as the controls, and a cohort of 20 infertile women, 10 of whom had recurrent implantation failure (RIF), and another 10 had unexplained infertility (UE). Using amplicon sequencing, which employs PCR to create sequences of DNA called amplicon, we compared the diversity, structure, and composition of faecal and vaginal bacteria of the controls with that of the infertile cohort. Of note, we could only sequence 8 vaginal samples in each group (n = 24/31).

**Result:**

Compared with the controls, α-diversity and β-diversity of the gut bacteria among the infertile groups differed significantly (*p* < 0.05). Taxa analysis revealed enrichment of Gram-positive bacteria in the RIF group, whereas Gram-negative bacteria were relatively abundant in the UE group. Strikingly, mucus-producing genera declined in the infertile cohort (*p* < 0.05). *Hungatella, *associated with trimethylamine N-oxide (TMAO) production, were enriched in the infertile cohort (*p* < 0.05). Vaginal microbiota was dominated by the genus *Lactobacillus,* with *Lactobacillus iners AB-1 *being the most abundant species across the groups. Compared with the infertile cohort, overgrowth of anaerobic bacteria, associated with vaginal dysbiosis, such as *Leptotrichia *and *Snethia*, occurred in the controls.

**Conclusion:**

The gut microbiota had little influence on the vaginal microbiota. Gut dysbiosis and vaginal eubiosis occurred in the infertile women, whereas the opposite trend occurred in the controls.

**Supplementary Information:**

The online version contains supplementary material available at 10.1186/s12905-022-01681-6.

## Introduction

Infertility, defined as a failure to conceive after 1 year of appropriately timed unprotected intercourse, is a distressing and costly reproductive disorder [1]. Some couples are diagnosed as having unexplained infertility (UE) because the underlying mechanism(s) remain undefined even after assessment of ovulatory function, tubal patency, and sperm parameters [2]. Frustratingly, manyinfertile couples undergo multiple unsuccessful assisted reproduction technology (ART) cycles (i.e., IVF and/or intracytoplasmic sperm injection (ICSI)) and are thus diagnosed as having repeated implantation failure (RIF) [2–4]. Yet another subgroup of infertile couples, diagnosed as recurrent pregnancy loss (RPL), exists that conceive several times (≥ 3), but miscarriage occurs each time before gestational week 28, although controversies exist on its definition [5, 6]. It has been argued that RIF and RPL represent the same condition spectrum [7]*.*

Pathologies of theseconditions converge on mechanisms by which the embryo fails to implant in the uterus [2, 3, 7, 8]. Implantation failure involves a triumvirate of a poor quality embryo and an unreceptive endometrium and an ill-timed embryo-endometrium interaction [3]. Although many systemic factors that disrupt implantation such as steroidal hormonal imbalance, thrombotic abnormalities, hyperhomocysteinemia, and immune dysfunctionshave been identified, much remains recondite [2, 3, 9].

The role of female lower reproductive tract bacteria has been shown in implantation failure [10–17]. By contrast, the role of gut bacteria in implantation failure remains barely explored. The gut bacteria, the densest and most diverse bacterial communities of the body, impact distal organs [18, 19]. They could impact implantation failure through the gut-reproductive tract microbiota axis. The gut bacteria exert a profound influence on the immune system, hormonal homeostasis, and the coagulation system.—all of which are known to be involved in embryo implantation [20–22].

Hence, we investigated whether gut dysbiosis occurs in women with implantation failure, and if so, whether it accompanies vaginal dysbiosis. To this end, by using 16S rRNA gene sequencing, we compared the diversity, structure, and taxonomic composition of the faecaland vaginal microbiota of fertile women with that of infertile women with a history of RIF and UE.

## Materials and methods

### Study participants

Fertile and infertile women,referred to Akanksha Hospital and Research Institute between September 2018 and February 2019, were recruited and divided into three groups: the control, RIF, and UE groups. The RIF group’s inclusion criteria were women who could not conceive after ≥ 2 fresh IVF-embryo transfer cycles/ICSI, or had ≥ 3 consecutive miscarriages [4, 5]. UE was diagnosed if a cause remains undefined after our routine fertility tests with the following criteria: infertility of more than 1 year, normospermic male partner, normal menstrual rhythm with regular ovulation, bilateral tubal patency verified through the hysterosalpingogram or laparoscopy, and normal hormonal tests (i.e., thyroid, prolactin, AMH) [23, 24]. Exclusion criteria includeddiabetes, polycystic ovary syndrome and endometriosis, diarrhoea, ongoing pregnancy, addiction (e.g., drugs, alcohol, tobacco etc.) and the use of antibiotics within at least two weeks before sample collection.

### Ethical approval and consent to participate

The local Ethics Committee of Sat Kaival Hospital Pvt. Ltd (EC2013/053) approved the study. We performed all the sampling and experiments in accordance with institutional guidelines for research with human subjects. Participants gave their oral and written informed consent for the sample collections and microbiological analysis. We recorded and compared participants’ characteristics (Table 1).

**Table 1:** Study characteristics of the control, RIF, and UE groups. Data are expressed as mean ± SD or n/N (%). In the RIF group, two participants belonged to the RPL category. a. statistically significant difference between the CON and the RIF group; b. statistically significant difference between the RIF group and the UE group

Characteristics	Control	RIF	UE
	(n = 11)	(n = 10)	(n = 10)
Age	27.9 ± 3.8	34.5 ± 4.8^a^	30.8 ± 3.42
(years)			
BMI	21.62 ± 4.05	25.9 ± 3.31^a^	23.92 ± 3
(kg/m^2^)			
Duration of infertility	–	9.5 ± 3.6	6.8 ± 2.2
AMH	–	3.1 ± 1.6	2.9 ± 0.89
Prolactin	–	7.7 ± 4.3	17.8 ± 6.6^b^
TSH	–	3.4 ± 1.7	2.4 ± 1.28
Nulligravida	–	2 (20%)	–-
		8 (80%)	10 (100%)
Nullipara			
Dietary information	Vegetarians		
	5	4	9
	45%	40%	90%

### Sample collection

The faecal and vaginal samples were freshly and simultaneously collected. Participants collected the faecal samples in a sterile plastic container with a tight closing lid [25]. To collect the vaginal samples, using a sterile swab stick, clinicians thoroughly wiped the posterior fornix of the vagina of the participants [26]. These swabs were stored in sterile vials. Both types of samples were packaged and first placed in a frozen storage at − 20 °C in the hospital and later, within 24 h, transported on ice to be stored at − 80 °C at Gujarat Biotechnology Research Centre (GBRC) for analysis.

### DNA extraction

DNA extraction was performed from approximately 200 mg of faecal samples and ~ 1 ml of thoroughly vortexed swab sample using QIAamp DNA Stool Mini Kit according to the manufacturer’s instructions. Total DNA was eluted in 30 μL of AE buffer. DNA concentration was quantified fluorometrically with a Qubit 2.0 dsDNA HS Assay kit. DNA was stored at − 20 °C for further procedures.

### Library preparation and 16S rRNA sequencing

The V2–V3 hypervariable regions of the 16S rRNA gene were amplified using fusion primers, 101 F5′ACTGGCGGACGGGTGAGTAA 3′ and 518 R 5′CGTATTACCGCGGCTGCTGG 3′ [27, 28]. Amplicon libraries were purified using the Agencourt AMPure XP (Beckman Coulter). For quality control, we used Bioanalyzer with a DNA-HS assay kit. These libraries were quantified using Qubit fluorimeter v4.0 and were pooled into equimolar concentrations. Using 530 chip and 400 base pairs sequencing chemistry, clonal amplification (Emulsion PCR) sequencing was performed on the Ion GeneStudio™ S5 System.

### Bioinformatics and statistical analysis

#### Diversity

Microbial richness and diversity were evaluated by α-diversity (Chao1 and Shannon indices). The Kruskal–Wallis test was used to determine statistical differences between the groups.The Mann–Whitney U test was used to determine the influence of diets on α-diversity and β-diversity.Differences in microbial community structures between the groups were analysed using Principle Coordinate Analysis (PCoA)  on  Jaccard distances, and the statistical difference between the groups was calculated using non-parametric permutational multivariate analysis of variance (PERMANOVA).

QIIME2 software was used to calculate alpha and beta diversity indices. The demultiplexed sequences were uploaded to QIIME2 environment, and denoising was carried out using DADA2. Amplicon sequence variants (ASVs) were predicted at a minimum sampling depth of 25,000 for Gut datasets, and 9000 for the vaginal datasets.The predicted ASVs were taxonomically classified using the pre-trained classifier of the full 16S rRNA gene sequence of the SILVA database.

#### Taxonomic structure

Microbial composition was analysed to identify taxa with significantly different abundance between the groups (relative abundance > 0.001 and P < 0.05). Linear discriminant analysis effect size (LEfSe) method was employed to identify species with significant differences in abundance between the groups (|LDA|> 3 and P < 0.05) [29]. Kruskal–Wallis and Mann–Whitney U tests were used to find statistical differences between the groups at taxonomic levels using STAMP v2.1 software [30]. To determine statistical differences in subjects’ characteristics between the groups, we performed one-way ANOVA followed by post-hoc Tukey testing or Student's t-test. Continuous data are presented as mean ± standard deviation (SD) or frequencies (number and percentages), calculated using GraphPad Prism statistical software 6.0.

## Results

### Metagenomics findings of gut and vaginal bacteria

Next-Generation sequencing of 31 faecal and 24 vaginal samples created a total of 81, 70,754 reads with an average of 2, 63,572 and 1, 07, 03,828 reads with an average of 4, 45,992 per sample for faecal and vaginal samples respectively. Based on the results of the operational taxonomic units (OTUs) analysis, rarefaction curves show that the sequencing depth was adequate to analyse the gut and vaginal bacteria in the three groups (Additional file 1: Figs S1 (a), (b) and Figs S2 (a), (b)).

### Richness and diversity of gut bacteria

**Fig. 1:**
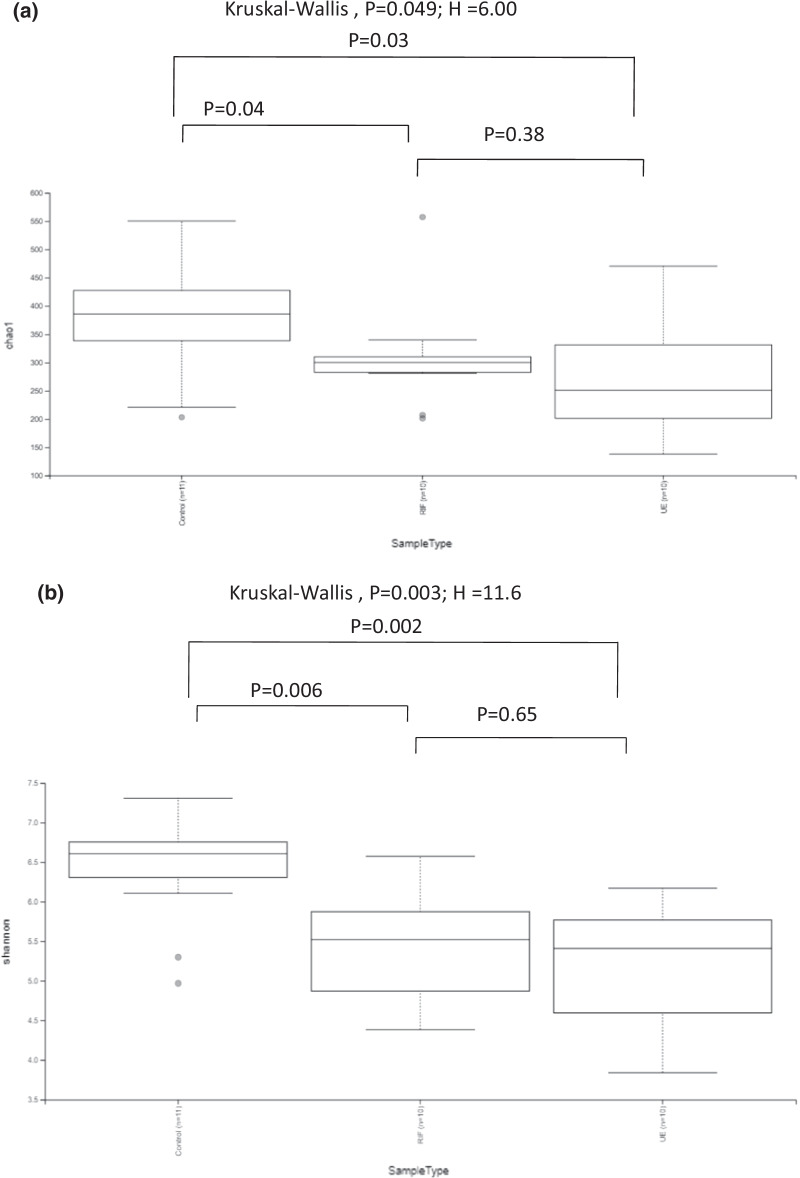
Box plots of α-diversity indices of the gut bacteria of the control (CON, N = 11), the RIF (RIF, N = 10), and the UE (UE, N = 10) groups: **a** Shannon and **b** Chao 1 indices

Diversity analyses revealed that the richness differed significantly between the three groups. We found that richness differed significantly between the three groups (Chao 1 index (Kruskal–Wallis test, *p* = 0.049)) (Fig. 1a).The controls had a significantly higher richness than the RIF group (pChao 1 = 0.04) and UE group (pChao 1 = 0.03). Richness was similar between the RIF and UE groups (pChao 1 = 0.75). We discovered that evenness differed significantly between the three groups (Shannon index (Kruskal–Wallis test, *p* = 0.003)) (Fig. 1b). Specifically, the controls had more evenness than the RIF (pShannon = 0.006) and the UE groups (pShannon = 0.002). In contrast, evenness was similar between the RIF and the UE groups (pShannon = 0.65). Interestingly, diet did not affect alpha diversity (pShannon = 0.165) (Fig. 3S).

**Fig. 2:**
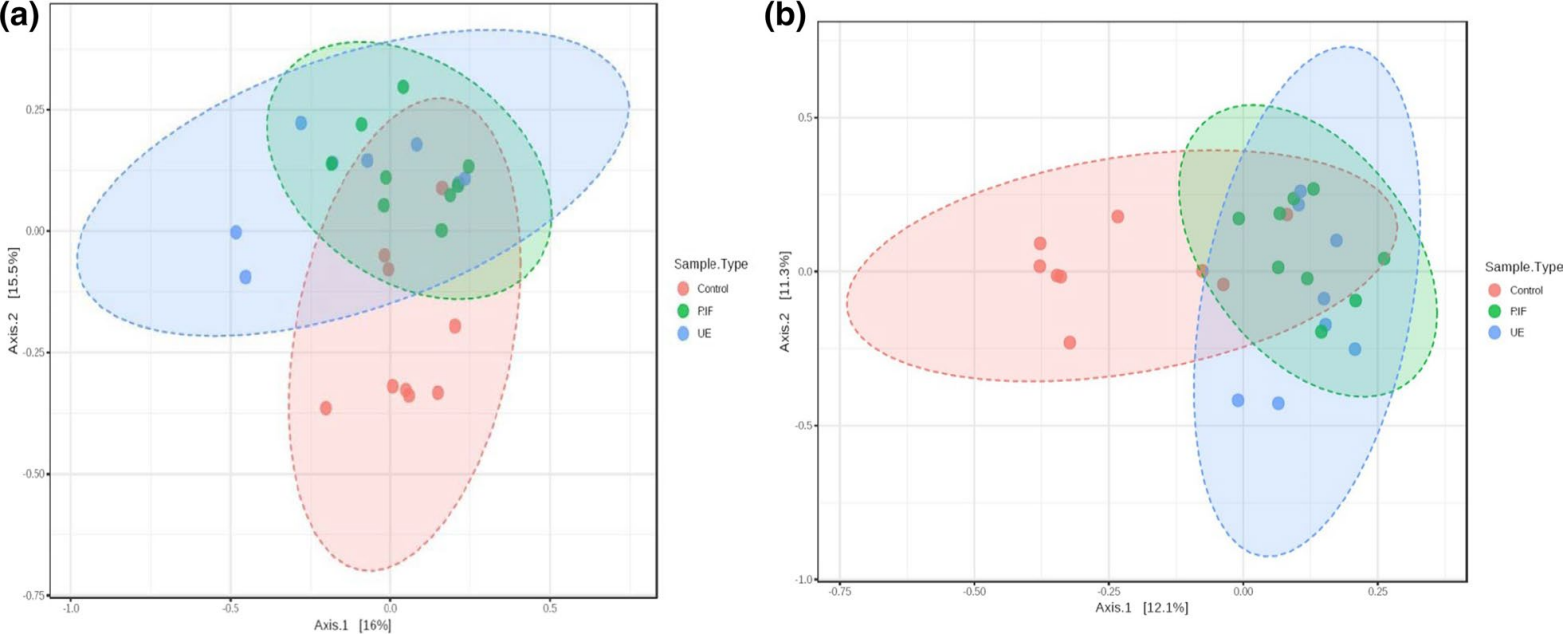
Differences in community composition (β-diversity) between the control (CON, Red, N = 11), the RIF (RIF, Green, N = 10), and the UE group (UE, Cyan N = 10) groups. Comparisons are based on the PCoA plots of Bray–Curtis (left) and Jaccard distances (right). Each principal coordinate axis represents the proportion of variance

Regarding the bacterial community structure differences, the PCoA plot of the Bray–Curtis and Jaccard dissimilarity showed that bacteria of the RIF and UE groups overlapped and that both groups differed markedly from the controls (Fig. 2a, b). In the PCoA plot based on Bray–Curtis distances, the first and second axes of the PCoA explained 21.5% of the total variance with a significant difference (PERMANOVA, P < 0.05, R2 = 0.12; Figs. 2a). Showing the similar clustering pattern, in the Jaccard based PCoA plot, the first and second axes explained 23.4% of the total variance with a significant difference (PERMANOVA, P < 0.05, R2 = 0.10; Fig. 2b).

### Taxonomic analysis of gut bacteria

After excluding the sequences that were present in less than 3%, we clustered the high-quality sequences into OTUs and assigned taxonomic identities. Consequently, we found 550 OTUs of 481 genera, 265 families, 156 orders, 68 classes, 715 species and 28 phyla. To evaluate the contribution of different taxa to diversity and composition, we calculated the relative abundance of taxa at the phylum, family and genus levels. Except at the genus level, we could not find statistically significant alteration of particular taxa at any other levels.

**Fig. 3:**
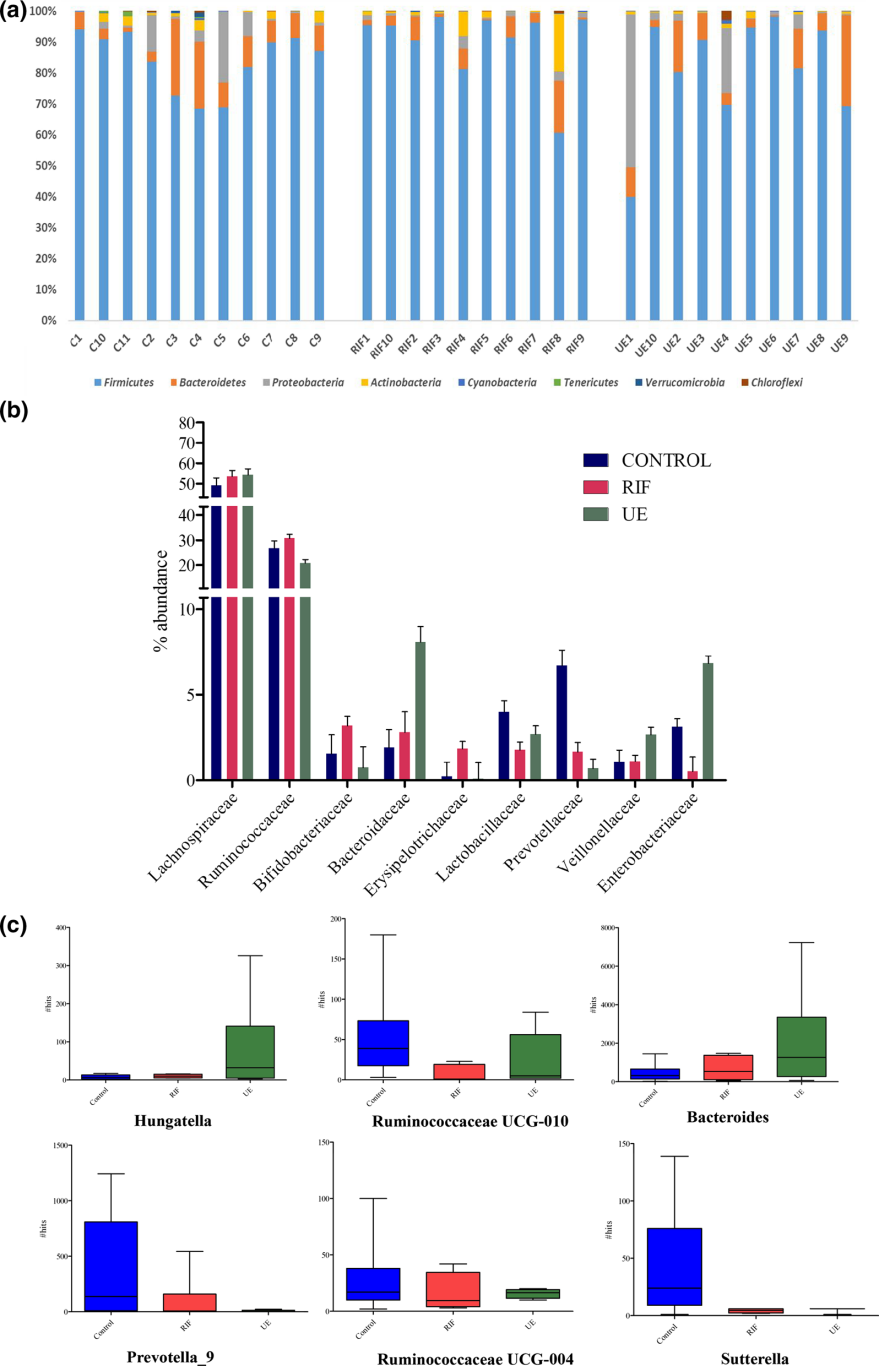
The bar chart shows the comparisons of relative abundances of top gut bacterial taxa between the control (CON, N = 11), the RIF (RIF, N = 10), and the UE (UE, N = 10) groups at **a** the phylum **b** family and **c** genus levels

Across all the participants, the four most abundant microbes were *Firmicutes* (85.10%), *Bacteroidetes* (7.70%), *Proteobacteria* (4.75%), and *Actinobacteria* (1.8%) (Fig. 3a).With a relative abundance of less than 1%, the remaining bacterial population belonged to four phyla, including *Verrucomicrobia, Tenericutes*, *Cyanobacteria*, and *Chloroflexi*.

Firmicutes were abundant in the RIF group than both the control (9% less) and UE groups (7% less). *Firmicutes* were more abundant (2%) in the UE group than the controls. *Bacteroidetes* were depleted (50% less) in the RIF group than the other two groups. In contrast, the control and UE groups had the same levels of *Bacteroidetes*. *Proteobacteria* were relativelyless abundant (4–7% less) in the RIF group than the control and UE groups (Fig. 3a)*.* Among the three groups, the UE group had the highest levels of *Proteobacteria*, almost twofold higher than the controls. *Actinobacteria* were depleted in the UE group (~ fourfold less) compared to the other two groups, with the RIF group showing the highest abundance amongst all the groups, with 2 fold more abundance than the control group (Fig. 3a).

The dominant bacterial families for all the subjects in the descending order of abundance were *Lachnospiraceae*, *Ruminococcaceae*, *Bifidobacteriaceae*, *Erysipelotrichaceae, Lactobacillaceae*, *Prevotellaacae*, *Vellinollacaea* and *Enterobacteriaceae* (Fig. 3b).The levels of *Lachnospiraceae* and *Ruminococcaceae* were similar between the three groups. Notably, the levels of *Lactobacillaceae* and *Prevotellaacae* families were highest in the controls as compared to the other two infertile groups. *Bacteroidaceae*, *Vellinollacaea* and *Enterobacteriaceae* were highest in the UE group compared to the RIF and control groups, while *Bifidobacteriaceae* and *Erysipelotrichaceae* families were highest in the RIF group as compared to the control  groups (Fig. 3b).

**Fig. 4:**
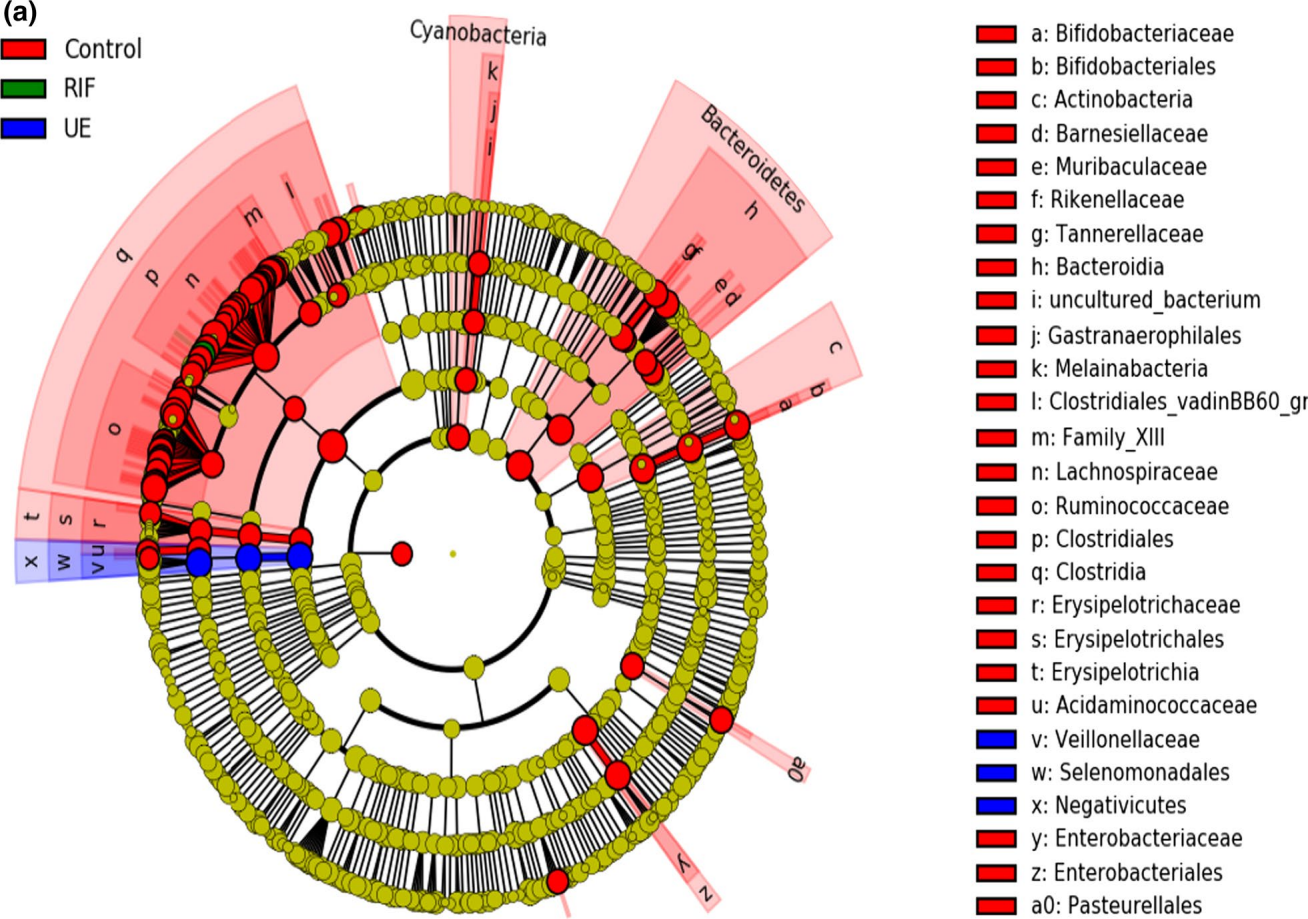

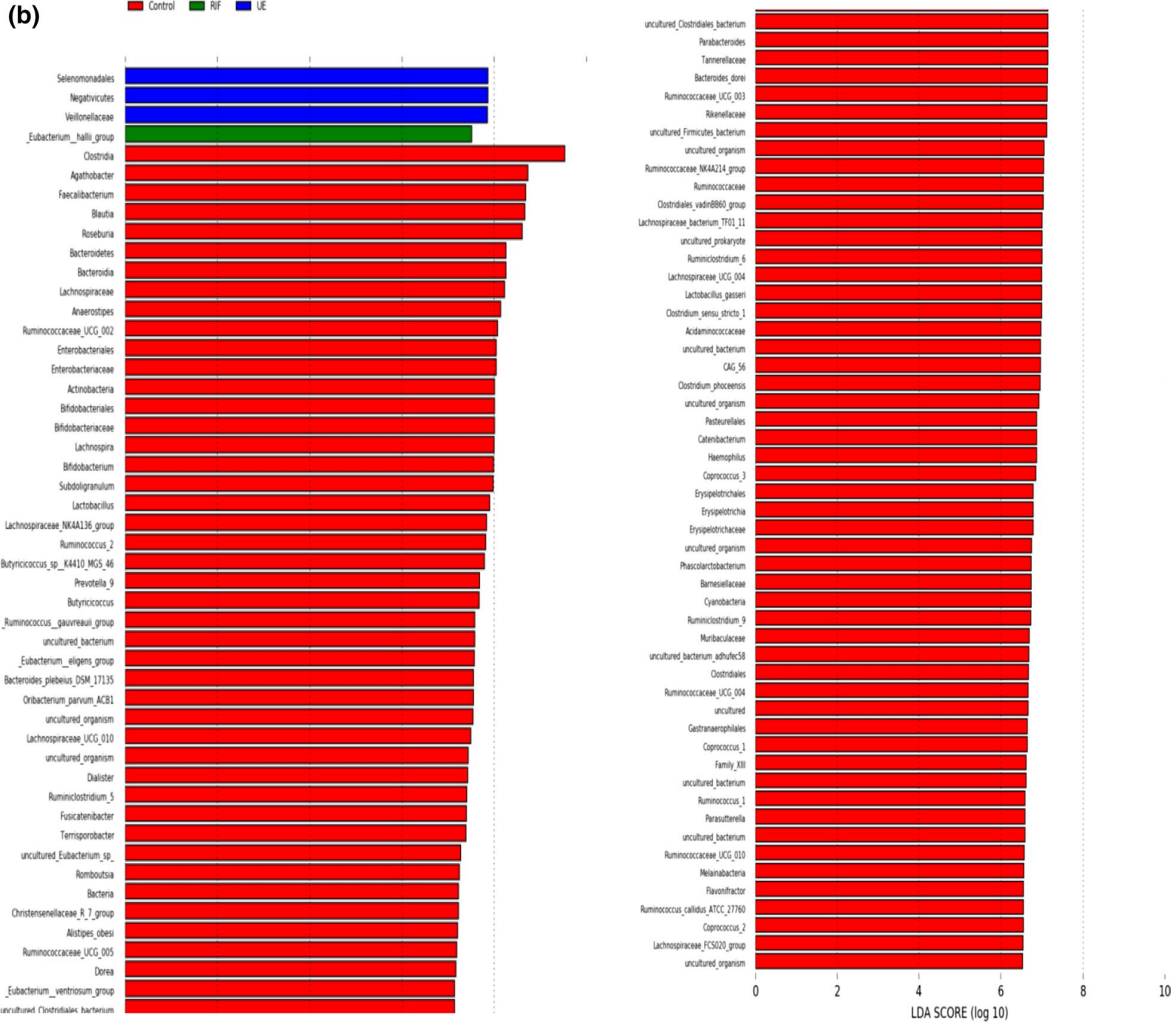
Distinct taxa of the gut bacteria determined by linear discriminant analysis effect size (LEfSe) analysis in the control (CON, N = 11), the RIF (RIF, N = 10), and the UE (UE, N = 10) groups. **a** The cladogram shows the taxa that were significantly elevated between the groups **b** Taxa with an LDA score significant threshold > 3 are shown (P < .05; LDA score 3)

We further determined statistical differences in the specific bacterial genera of the three groups. Among 481 genera, the 6 were statistically significantly (p < 0.05) different:*Bacteroides, Prevotella 9*, *Hungatella*, *RuminococcaceaeUCG-004, Ruminococcaceae UCG-010,* and *Sutterella. *Aside from *Bacteroides Prevotella 9*, the abundance of the other 5 genera, while statistically significant (p < 0.05), occurred in much lower proportions (< 1%). Notably, *Bacteroides* and *Hungatella* were more abundant in the infertile cohort, especially in the UE group, than in the controls (Fig. 3c).When wecompared the infertile group against the controls, we found that in the infertile cohort *Prevotella 9, Ruminococcaceae UCG-004, Ruminococcaceae UCG-010 *(p < 0.05) declined,whereas *Bacteroides, Dorea, oral clone FR58 *and *Peptoniphilus *increased (p < 0.05) (Fig. 4S)*.*

### LEfSe analysis

LEfSe analysis was performed to assess the differentially abundant communities in the three groups (Fig. 4a, b). In the controls, we observed diverse microbial communities with a high LDA score (Log10), with *Clostridia* showing the highest LDA score > 9(p < 0.05). In the RIF group, only *Eubacteriumhalli* showed the highest dominance with an LDA score > 7(p < 0.05). In the UE group, unconventional *Firmicutes* such as *Veillonellaceae*, *Selenomonadales*, of the class *Negativicutes, *showed the highest preponderance with an LDA score > 7 (p < 0.05).

### Metagenomics of vaginal bacteria

Across the 24 vaginal samples, we found 384 distinct species belonging to 301 different genera classified in 135 different families,distributed into 10 phyla. We compared taxa between the three groups. Given the small sample size, we could not detect a significant statistical difference between them.

### Taxonomic analysis of vaginal bacteria

**Fig. 5:**
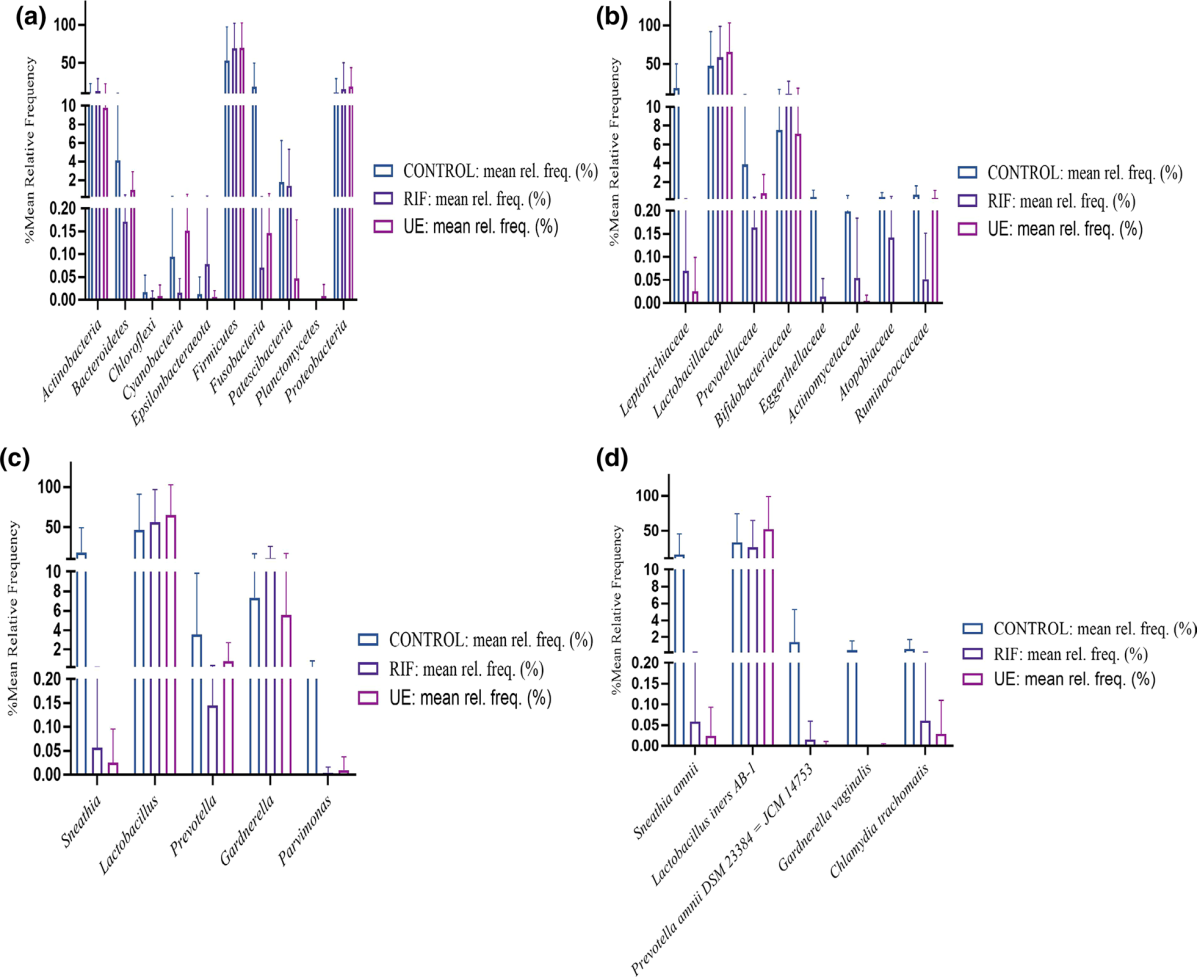
The bar charts show taxonomic comparisons of the vaginal bacteria between the control (CON, N = 8), the RIF (RIF, N = 8), and the UE (UE, N = 8) groups at **a** the phylum, **b** family **c** genus **d** species levels

In descending order, the dominant phyla, among the 10 detected phyla, included *Firmicutes, Fusobacteria*, *Proteobacteria, Actinobacteria, Bacteroidetes*, and *Patescibacteria* (Fig. 5a). Of these, *Firmicutes *accounted for the vast majority of the vaginal bacteria in all the groups, with both the RIF (69%) and UE (69.71%) groups showing similar relative abundance, which was higher than the controls (53%). *Fusobacteria* (18% vs. 0.07 vs. 0.14) and *Bacteriodetes* (4.1% vs. 0.17 vs. 0.92) were relatively more abundant in the controls than in the RIF and UE groups. *Proteobacteria* were marginally more abundant in both the RIF (15% vs.11%) and the UE (19% vs. 11%) groups compared to the controls (Fig. 5a).

The dominant families for all the groups in descending order of abundance were *Lactobacillaceae, Bifidobacteriaceae, Leptotrichiaceae,*and *Prevotellaceae* (Fig. 5(b)). Lactobacillaceae and *Bifidobacteriaceae *were present in all the groups*.* Reflecting this trend at the phylum level, levels of *Lactobacillaceae* were higher in all the groups, with UE (65.3%) and RIF (58.41%) women showing the highest levels compared with the controls (47.2%).

At the genus level, 5 genera were detected, of which three were present in all the groups (Fig. 5c). *Lactobacillus* was the most dominant genus among them, followed by *Gardnerella* and *Parvimonas*. *Gardnerella, Prevotella*, *Parvimonas,* and *Snaethia*were relatively more abundant in the controls compared to the infertile groups. Compared to the controls, *Lactobacillus *was relatively more abundant in the RIF and the UE groups.

At the species levels, *Sneathia ammni* (0.36%) was detected only in the control groups. In contrast, *Lactobacillus iners AB-1 *were present in all the groups, with descending order of relatively high abundance in the following manner: the UE group (62%), the controls (16%), and the RIF group (11.02%) (Fig. 5d).

###  LEfSe analysis

**Fig. 6:**
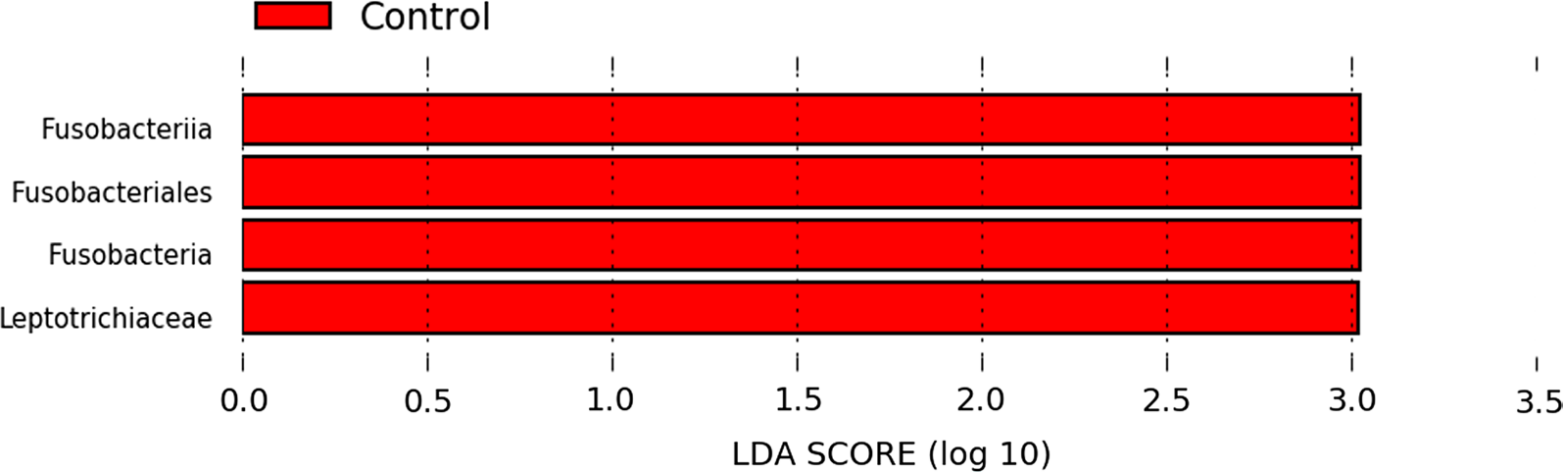
Differentially abundant vaginal bacteria between the control (CON, N = 8), the RIF (RIF, N = 8), and the UE (UE, N = 8) groups as determined by linear discriminant analysis effect size (LEfSe) analysis. Taxa with an LDA score significant threshold > 3 are shown (P < .05; LDA score 3)

We performed LEfSe analysis to assess the differentially abundant vaginal bacterial communities in the three groups. We could only find significant differences in the controls, with *Leptotrichia*, of the *Fusobacteria*, showing LDA (Log10) score > 3 (p < 0.05) Fig. 6.

### Alterations of the genus lactobacillus species

**Fig. 7:**
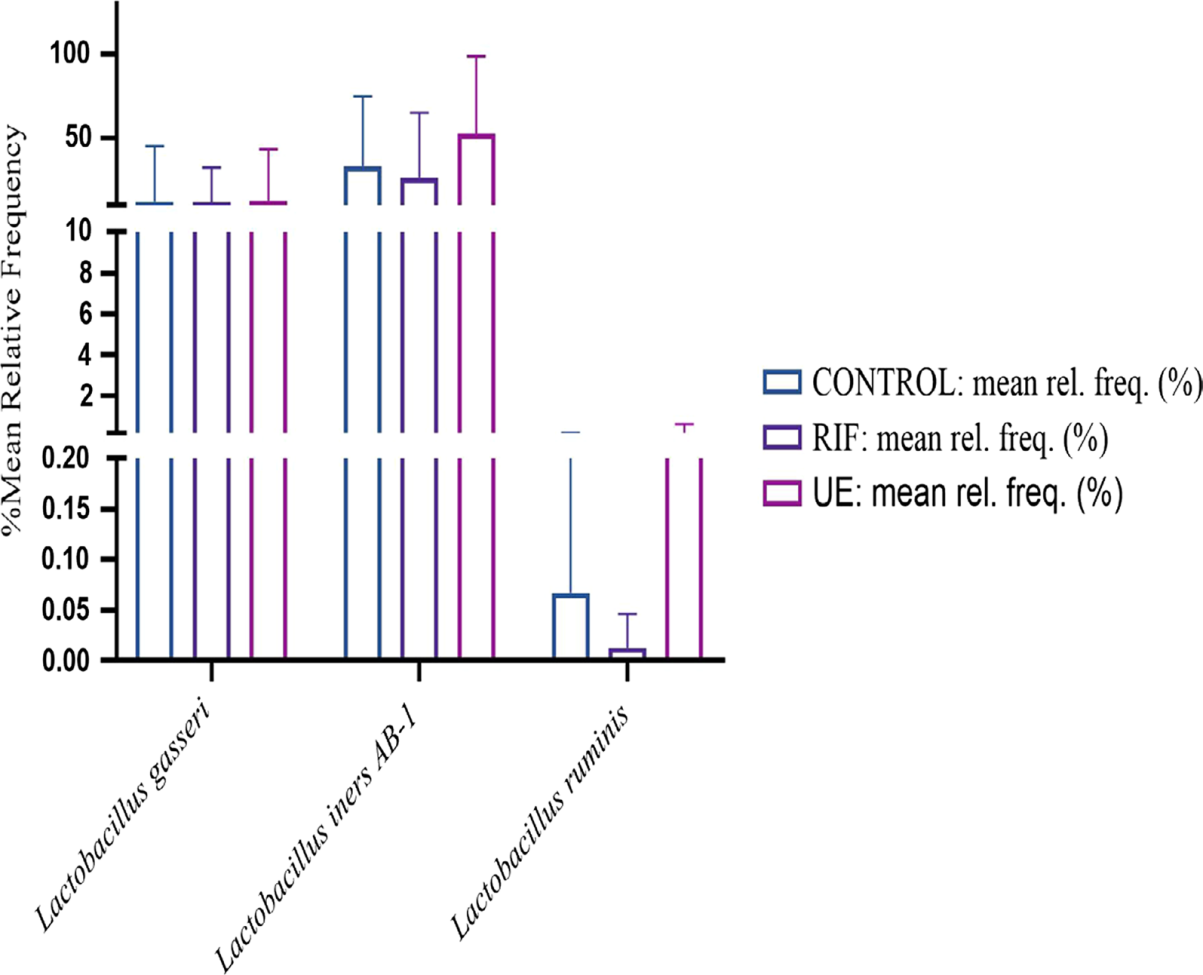
The bar charts show taxonomic comparisons of different *Lactobacillus *spp. of the vagina between the control (CON, N = 8), the RIF (RIF, N = 8), and the UE (UE, N = 8) groups

Within the genus of *Lactobacillus*, 9 species were identified. Of them, *L. gasseri, L. ruminis, and L. iners AB-1 *were found in all the groups, with *Lactobacillus iners AB-1* being the most abundant species (Fig. 7). Among these, *L. jensenii* and *L. vaginalis* were only detected in the UE group, while *L. reuteri *was unique to the RIF group. *L. equicursoris, L. fermentum* and *L. salivarius* were unique to the controls.

## Discussion

We compared the gut-vaginal microbiota axis of fertile women with that of women diagnosed with RIF and UE.The core findings include (i) the infertile groups had gut dysbiosis as evident by low α-diversity indices and beta diversity metrics; (ii) the gut microbial composition of the RIF and UE groups differed, with a set of Gram-positive taxa, being dominant in the former group and a set of Gram-negative bacteria, being dominant in the latter group; (iii) butyrate-producing genera such as *Prevotella *declined in the infertile cohort; (iv) elevated levels of the genus *Hungatella *occurred in the infertile cohort; and (v) the infertile cohort had a comparatively healthy vaginal microbiota. Of note, Azpiroz et al. recently reported similar findings in a large cohort of infertile women [31].

Gut microbial richness and diversity,defined by α-diversity indices, declined amongst the infertile groups, with the highest decline in the UE group. Notably, reduced *α*-diversity indicates low-grade inflammatory disorders such as inflammatory bowel disease and metabolic disorders [32–34]. Gut dysbiosis was also reflected in beta diversity indices, suggestinga distinct bacterial composition between the infertile cohort and the controls.

Taxa analysis showed that at the genus level, a relative decline in the abundance of *Prevotella* (phylum *Bacteroidetes*) and an increase in the abundance of *Bacteroides* (phylum *Bacteroidetes*) commonly occurred in the infertile groups. Since *Prevotella *builds the protective gut *mucosal* barrier of mucin gel from short-chain fatty acids (SCFAs) such as butyrate and since *Bacteroides* prevents mucin synthesisby producing metabolites such as succinate, acetate, and propionate, this suggests weakened mucosal protectionin the infertile cohort [35]. In keeping with this, other butyrate-producing genera that included Ruminococcaceae UCG-004 and Ruminococcaceae UCG-010, of the *Ruminococcaceae*, family and the genus *Sutterella* (Phylum *Proteobacteria*) declined in the infertile cohort. [36–38]. Conversely, beneficial commensal *Clostridia* were relatively enriched in the controls [39].

When the mucus barrier erodes, the gut bacteria and other microbe-associated molecular patterns (MAMPs) come in contact with toll-like receptors (TLRs), located in the gut epithelial cells such as the Paneth cells [40–42]. TLRs recognise microbes and MAMPs, and subsequently elicit an immune response, leading to localised and systemic inflammation. This suggests the involvement of gut dysbiosis-induced systemic inflammation in implantation failure [35, 40–42]. We proffer the following mechanistic hypotheses to explain how the gut dysbiosis in the infertile groups causes implantation failure by separate mechanisms that promote systemic inflammation.

We posit that gut dysbiosis–induced metabolic dysregulation plays a role in RIF. At the phyla level,the abundances of *Bacteroidetes* and *Proteobacteria* were lower. By contrast, the levels of *Firmicutes *and *Actinobacteria* were higher in the RIF group compared with the other two groups–indicating an obesity-associated microbiota profile [43]. Indeed, obesity has been linked to an increase in *Firmicutes-* to- *Bacteroidetes* (F/B) ratio [43, 44]. In fact, the RIF group’s mean BMI, highest among the three groups, was in the obesity range (obesity ≥ 25 kg/m^2^ for Asian Indians [45]), which confirms the fact that obesity is a risk factor for RIF [46].

The Clostridium XIVa cluster, of the *Firmicutes* phylum, whose members comprise flagellated bacteria with a tendency to colonise mucus, play a critical role in metabolic dysregulation such as obesity [37, 44, 47]. Indeed, a trend towards an increase in the relative abundance of *Firmicutes *genera in this cluster such as *Lachnoclostridium,* *Dorea,* *Ruminococcus 2,* and *Eubacterium* was duly noted in the RIF group [47]. LEfSe analysis found that *Eubacteriumhalli*, a member of this cluster, previously found to be elevated in human obesity, is a RIF biomarker [44, 48]. Strikingly, the RIF group had the highest levels of the *Erysipelotrichaceae* family, which was almost absent in the other groups. Elevated levels of *Erysipelotrichaceae* have been linked to human obesity and have been correlated with elevated levels of Tumor Necrosis Factor-alpha (TNF-α), a pro-inflammatory cytokine involved in obesity-linked insulin resistance [49]. Tellingly, a high relative abundance of *Firmicutes *has been shown to correlate with increased levels of peripheral TNF-α [50]. A rodent study found that a high-fat diet first increased the phylum *Firmicutes,*corresponding with the changes of Panethcell-antimicrobial peptides, which was later followed by the elevations of circulating inflammatory cytokines, including TNF-*α,* thus establishing causality between the phylum *Firmicutes* and TNF-α [51]*.*

We postulate that the phylum *Firmicutes* generates TNF-α-driven systemic inflammation and consequent insulin resistance may cause RIF. Strikingly, investigators showed elevated TNF-α/IL-10 ratio correlates with an increased risk of IVF failure [52].Chan et al*.* found that insulin resistance reduces implantation rate in in vitro maturation-in vitro fertilization-embryo transfer cycle [53]. Metformin, known to reduce the F/B ratio, has been shown to increase the pregnancy rate in IVF repeaters without polycystic ovary syndrome [54, 55]. Investigators showed an arginine-rich diet, known to reduce obesity and increase insulin sensitivity, corrects the elevated F/B ratio, and increases embryo survival [56].

The most striking phyla level change in the UE group involved depletion of *Actinobacteria* and abundance of *Proteobacteria*, the pro-inflammatory phylum, comprising common pathogens (e.g., *Escherichia, Salmonella*) [57]. This suggests a critical role of *Proteobacteria *phyla in UE. This concurs with the fact that *Bifidobacterium*, a genus of the depleted phyla *Actinobacteria,* inhibits gut pathogens [58]. Unsurprisingly, the UE group had the highest enrichment of pathogenic Gram-negative families, whose outer membrane contains lipopolysaccharides (LPS) [59]. These bacterial families included: *Bacteroidaceae* (phylum *Bacteroidetes*), *Veillonellaceae* (phylum *Firmicutes*), and *Enterobacteriaceae* (phylum *Bacteroidetes*). Cogently, LEfSe analysis revealed members of the *Negativicutes* class—such as *Veillonellaceae*, *Selenomonadales*, which are atypical gram-negative *Firmicutes*, which possess LPS in the outer membranes—were biomarkers of UE [60].

The abundance of *Negativicutes *has been linked with an increase in the systemic levels of IL-6, the pro-inflammatory cytokine [61]. Along this line, enrichment of other Gram-negative species has been shown to increase the plasma levels of IL-6 [61–63]. LPS of Gram-negative species, a pro-inflammatory endotoxin, bind to TLR-4 in the gastrointestinal mucosa, triggering an inflammatory cascade that causes localised NF-κB activation, which leads to thesystemic secretion of IL-6 [42].

Taken together, we proffer that in the setting of the porous mucosal barrier, the overload of Gram-negative bacteria activates the gut innate immune system, generating IL-6-driven systemic low-grade inflammation, ultimately leading to UE. Indeed, Demiret al*.* found higher serum IL-6 levels, but not TNF-α levels, in women with UE [64]. Since elevated IL-6 levels impairs various aspects of reproductive physiology, including LH secretion, LH-induced ovulation, and FSH-stimulated E2 and progesterone release, the gut bacteria-induced higher IL-6 levels may thus cause UE through these mechanisms [64].

In the infertile cohort, fascinatingly, higher levels of *Hungatella*, producers of trimethylamine N-oxide (TMAO), which enhances thrombotic potential through platelet hyperreactivity, were found than inthe controls [65, 66]. This raises the possibility that an overactive  system is a common mechanism of implantation failure. Since levels of *Hungatella *were highest in the UE group, this indicates an important role of thrombosis in UE. Indeed, Azem et al. found inherited thrombophilia plays a role in repeated IVF failures, particularly in the subgroup with UE [67].

Regarding the landscape of the vaginal microbiota, consistent with previous research, *Firmicutes*, mainly *Lactobacilli* spp., constituted the bulk of total bacteria across the groups [68, 69]. By lowering the vaginal PH < 4 through lactic acid production, generating bacteriocins and hydrogen peroxide (H_2_O_2_), or acting as a competitive inhibitor, *Lactobacilli* spp*.* protect the vagina from opportunistic pathogens [70, 71]. Of the nine detected *Lactobacilli* spp., three species dominated the vaginal microbiota across the groups: *L. iners, L. gasseri*, and *L. ruminis,* with *L. iners* being the most abundant, suggesting the existence of community state type 3 (CST 3) of the five human vaginal microbial communities (HVMC) as classified by Ravel et al. (2011) [72].

Furthermore, data showed that the vaginal bacterial community was less diverse than in the gut.  analysis found that the RIF group had the lowest microbial diversity of the three groups, suggesting a healthy vaginal microbiota in the RIF group. This chimes with the finding that BMI negatively correlates with vaginal dysbiosis [73]. By contrast, the highest microbial diversity in the control group suggests the presence of vaginal dysbiosis. Indeed, LEfSe analysis found *Leptotrichia*, an opportunistic pathogen of the female urogenital tract (phylum *Fusobacteria*) in this group [74]. In line with this, other pathogenic genera such as *Gardnerella, Prevotella*, and *Snaethia *were relatively more abundant in the controls compared to the infertile groups [74, 75].

## Conclusion

In sum, this study illuminated gut and vaginal bacterial communities’ landscape, both at broader and finer levels, in both infertile and fertile women and offers conjectures to explain the data. We discovered that the infertile cohort had gut dysbiosis but not vaginal dysbiosis. The study has laid the foundation of research on the link between the gut microbiota, the gut-reproductive microbiota axis, and implantation failure, which can lead to microbiota-based diagnostic tools and therapeutic strategies.

## Limitations

Our study has a few limitations. First, since it is an underpowered single-center study, multi-center longitudinal studies with a large sample size are needed. Second, although we suggested the mechanistic hypotheses, we did not measure alterations in the immune system, hormones, platelet parameters and bacterial metabolic products such as short-chain fatty acids. Third, owing to the limited resolution of the 16S rRNA-sequencing technique, we could not identify what specific bacterial species or strains were involved. Finally, the functional significance of many species such as *peptoniphilus *remains undetermined in our analysis as the literature is scant on these genera. Hence, future investigations should address these shortcomings.

## Supplementary Information

**Additional file 1.** **Supplementary figures. Figure 1S.** Rarefaction analysis of Gut and Vaginal Microbial diversity of the controls, RIF and UE groups. (A) Rarefaction curve of α-diversity of the gut bacteria in control (CON, N = 11), RIF (RIF, N = 10), and the UE (UE, N=10) groups (B) Rarefaction curve of α-diversity of the vaginal bacteria in control (CON, N = 8), RIF (RIF, N = 8), and the UE (UE, N=8) groups. The x-axis shows the number of sequences per sample, and the y-axis shows the rarefaction measure. When the curve plateaus, it shows that the sequencing data volume is sufficient to reveal most of the microbial information in the sample. The values of the y-axis reflect the community diversity of microbiota. The sequence number in the chart shows the sequence number of the sample. OTU, operational taxonomic unit. **Figure 2S.** Rarefaction analysis of microbiome diversity sequences per sample: (a) gut (n = 31) and (b) vaginal samples (n = 24) (Operational taxonomic units (OTUs) for each sample at 97% of similarity). Each graph represents mean (column) and SD (bars). **Figure 3S.** Influence of diet on α-diversity between vegetarian (n = 21) and non–vegetarian participants (n = 10). No significant difference in α-diversity between these groups was found (P > 0.05, the Mann-Whitney U test). **Figure 4S.** The bar chart shows taxonomic comparisons of the gut bacteria between the controls (CON, N = 11) and the infertile cohort (the RIF plus UE groups, N = 20) at the genus level with statistical significance values (P > 0.05, Mann-Whitney U test).

## Data Availability

The 16S rRNA gene sequencing data for all the gut and vaginal microbiota samples analyzed in this study have been deposited with the National Center for Biotechnology Information (NCBI): reference number PRJNA7020230.

## Abbreviations

AMH: Anti-Müllerian hormone
ASV: Amplicon sequence variants
BMI: Body- mass index
ET: Embryo transfer
F/B ratio: Firmicutes-to-Bacteroidetes ratio
ICSI: Intracytoplasmic Sperm Injection
IL-6: Interleukin 6
IL-10: Interleukin 10
IVF: In vitro fertilization
*L. delbrueckii*: *Lactobacillus delbrueckii*
*L. equicursoris*: *Lactobacillus equicursoris*
*L. fermentum*: *Lactobacillus fermentum*
*L. gasseri*: *Lactobacillus gasseri*
*L. iners*: *Lactobacillus iners*
*L*. *jensenii*: *Lactobacillus* *jensenii*
*L. reuteri*: *Lactobacillus reuteri*
*L. ruminis*: *Lactobacillus ruminis*
*L. salivarius*: *Lactobacillus salivarius*
*L. vaginalis*: *Lactobacillus vaginalis*
LDA: Linear discriminant analysis
LEfSe: Linear discriminant analysis effect size
LPS: Lipopolysaccharides
MAMPs: Microbe-associated molecular patterns
OTU: Operational taxonomic units
PCR: Polymerase chain reactions
PERMANOVA: Permutation multivariate analysis of variance
PCoA: Principle Coordinate Analysis
RIF: Recurrent implantation failure
SCFA: Short-chain fatty acids
16S rna: 16S ribosomal RNA
TLRs: Toll-like receptors, TMAO Trimethylamine N-oxide
TNF-α: Tumour Necrosis Factor-α
TSH: Thyroid stimulating hormone
UE: Unexplained infertility
