# Supplementary material for: Distinct gut and vaginal microbiota profile in women with recurrent implantation failure and unexplained infertility
Source: BMC Womens Health. 2022 Apr 12;22:113. doi: 10.1186/s12905-022-01681-6 (PMC9004033; doi:10.1186/s12905-022-01681-6)
Supplement: Supplementary file 1 — Additional file 1. Supplementary figures. Figure 1S. Rarefaction analysis of Gut and Vaginal Microbial diversity of the controls, RIF and UE groups. (A) Rarefaction curve of α-diversity of the gut bacteria in control (CON, N = 11), RIF (RIF, N = 10), and the UE (UE, N=10) groups (B) Rarefaction curve of α-diversity of the vaginal bacteria in control (CON, N = 8), RIF (RIF, N = 8), and the UE (UE, N=8) groups. The x-axis shows the number of sequences per sample, and the y-axis shows the rarefaction measure. When the curve plateaus, it shows that the sequencing data volume is sufficient to reveal most of the microbial information in the sample. The values of the y-axis reflect the community diversity of microbiota. The sequence number in the chart shows the sequence number of the sample. OTU, operational taxonomic unit. Figure 2S. Rarefaction analysis of microbiome diversity sequences per sample: (a) gut (n = 31) and (b) vaginal samples (n = 24) (Operational taxonomic units (OTUs) for each sample at 97% of similarity). Each graph represents mean (column) and SD (bars). Figure 3S. Influence of diet on α-diversity between vegetarian (n = 21) and non–vegetarian participants (n = 10). No significant difference in α-diversity between these groups was found (P > 0.05, the Mann-Whitney U test). Figure 4S. The bar chart shows taxonomic comparisons of the gut bacteria between the controls (CON, N = 11) and the infertile cohort (the RIF plus UE groups, N = 20) at the genus level with statistical significance values (P > 0.05, Mann-Whitney U test). [file 12905_2022_1681_MOESM1_ESM.pptx]

## Slide 1
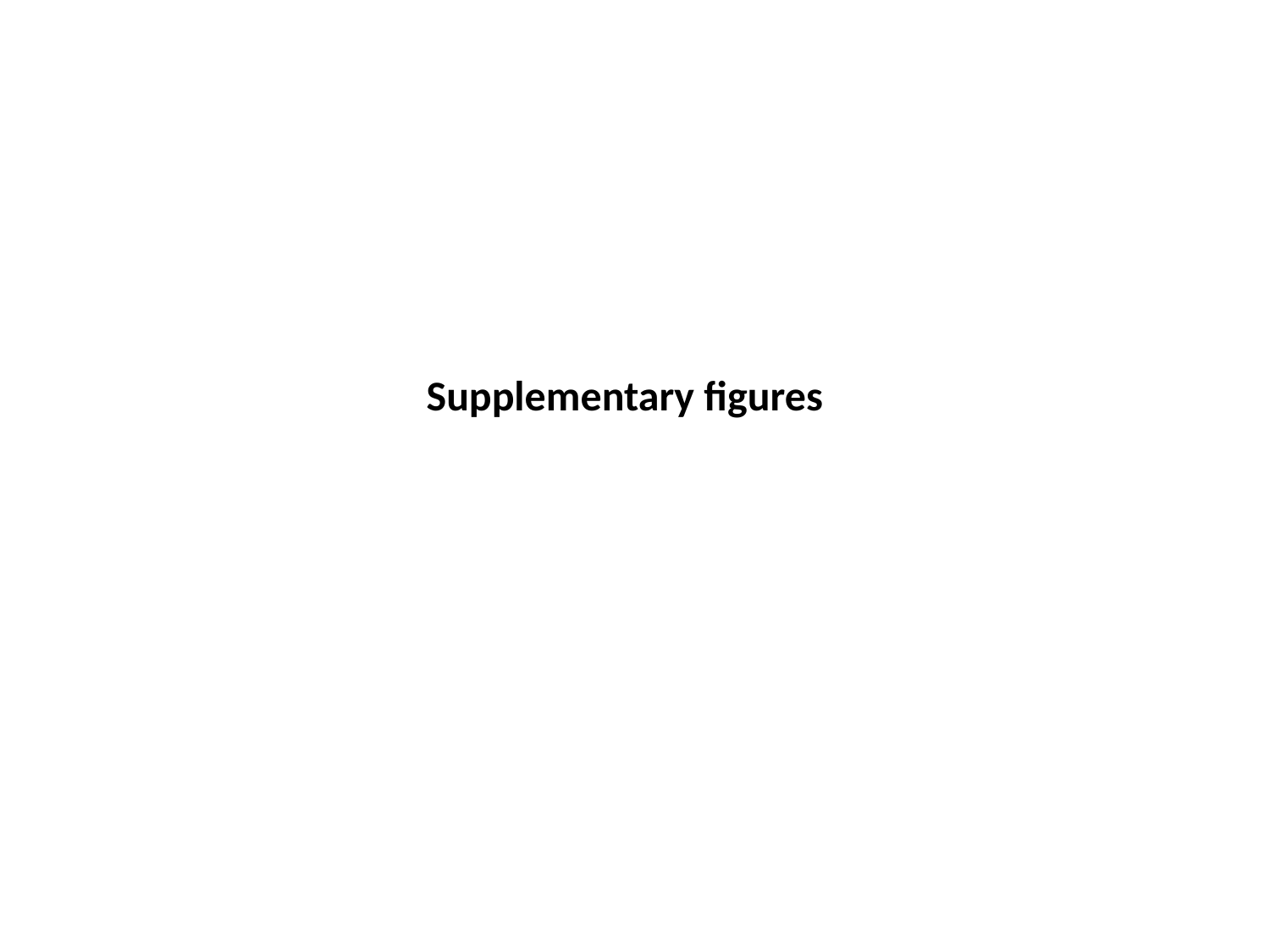

# Supplementary figures

## Slide 2
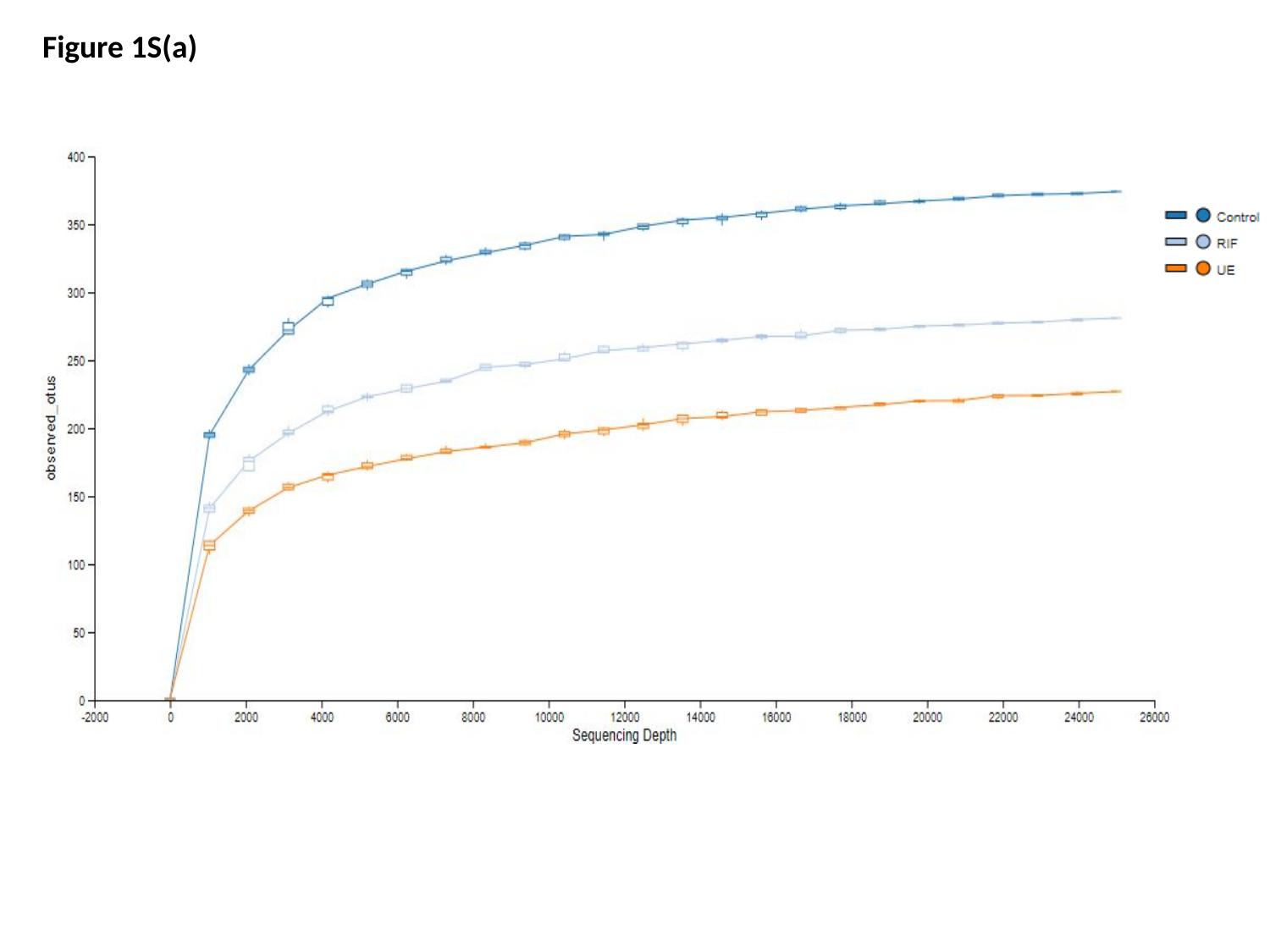

Figure 1S(a)

## Slide 3
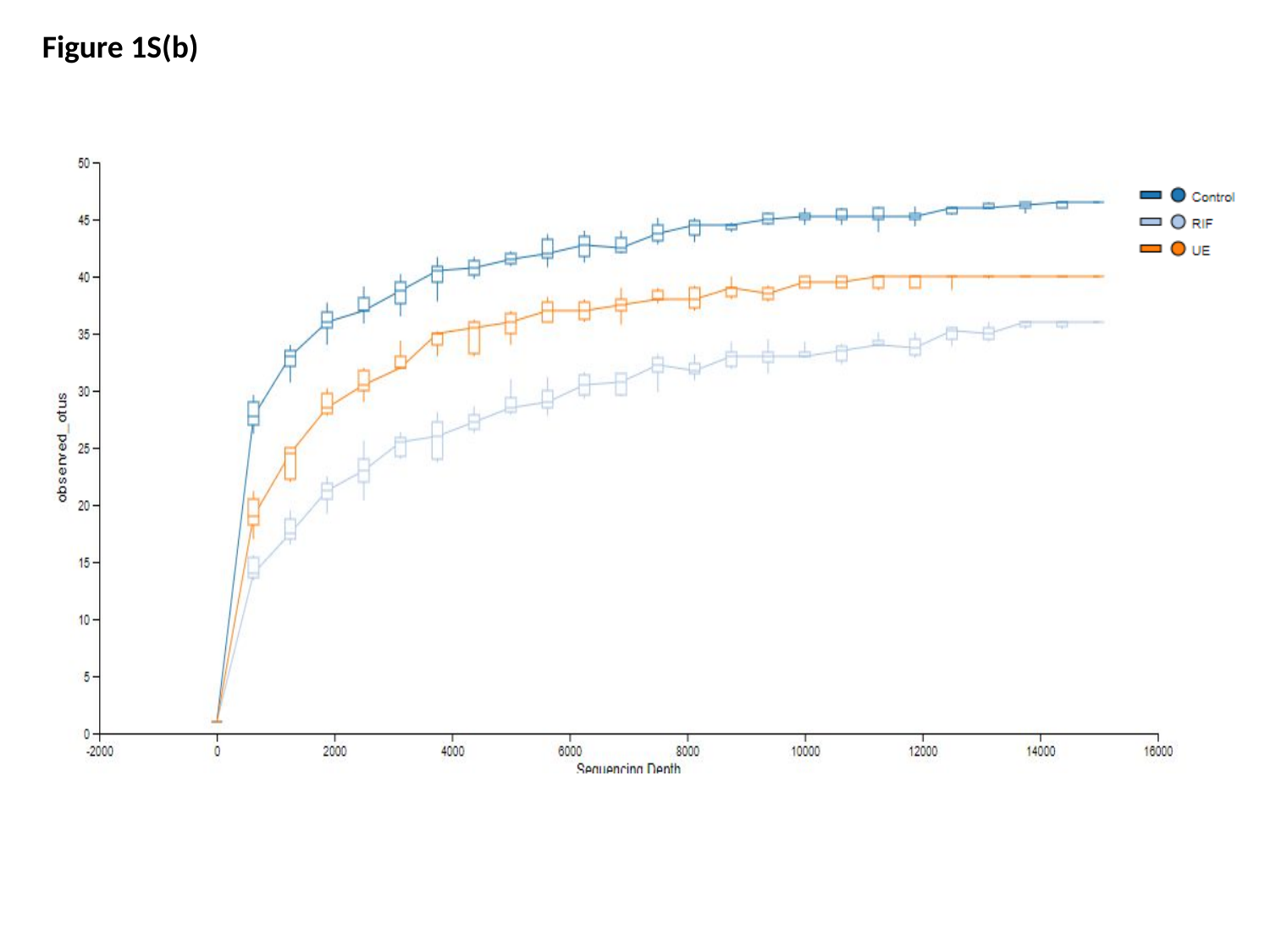

Figure 1S(b)

## Slide 4
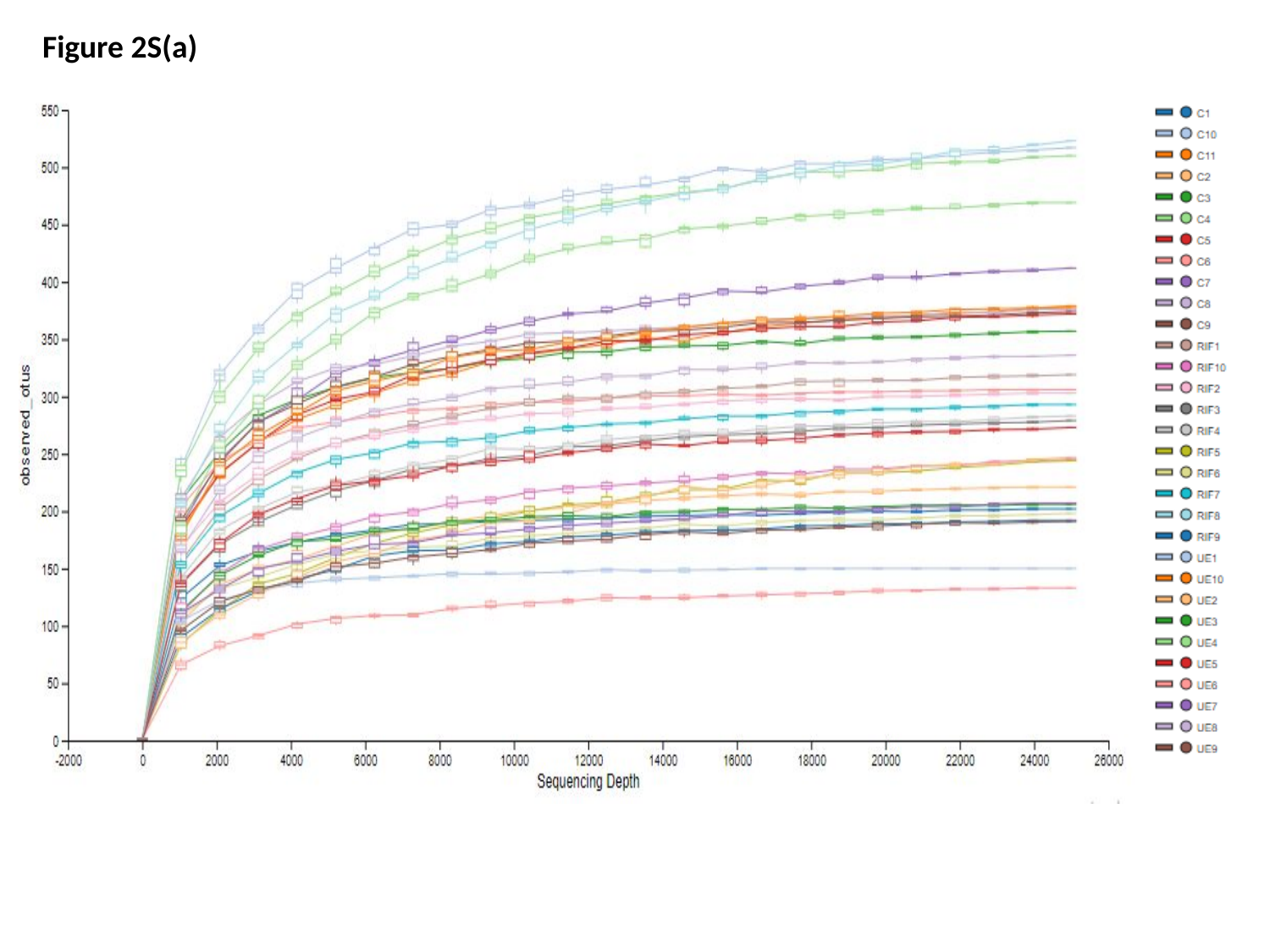

Figure 2S(a)

## Slide 5
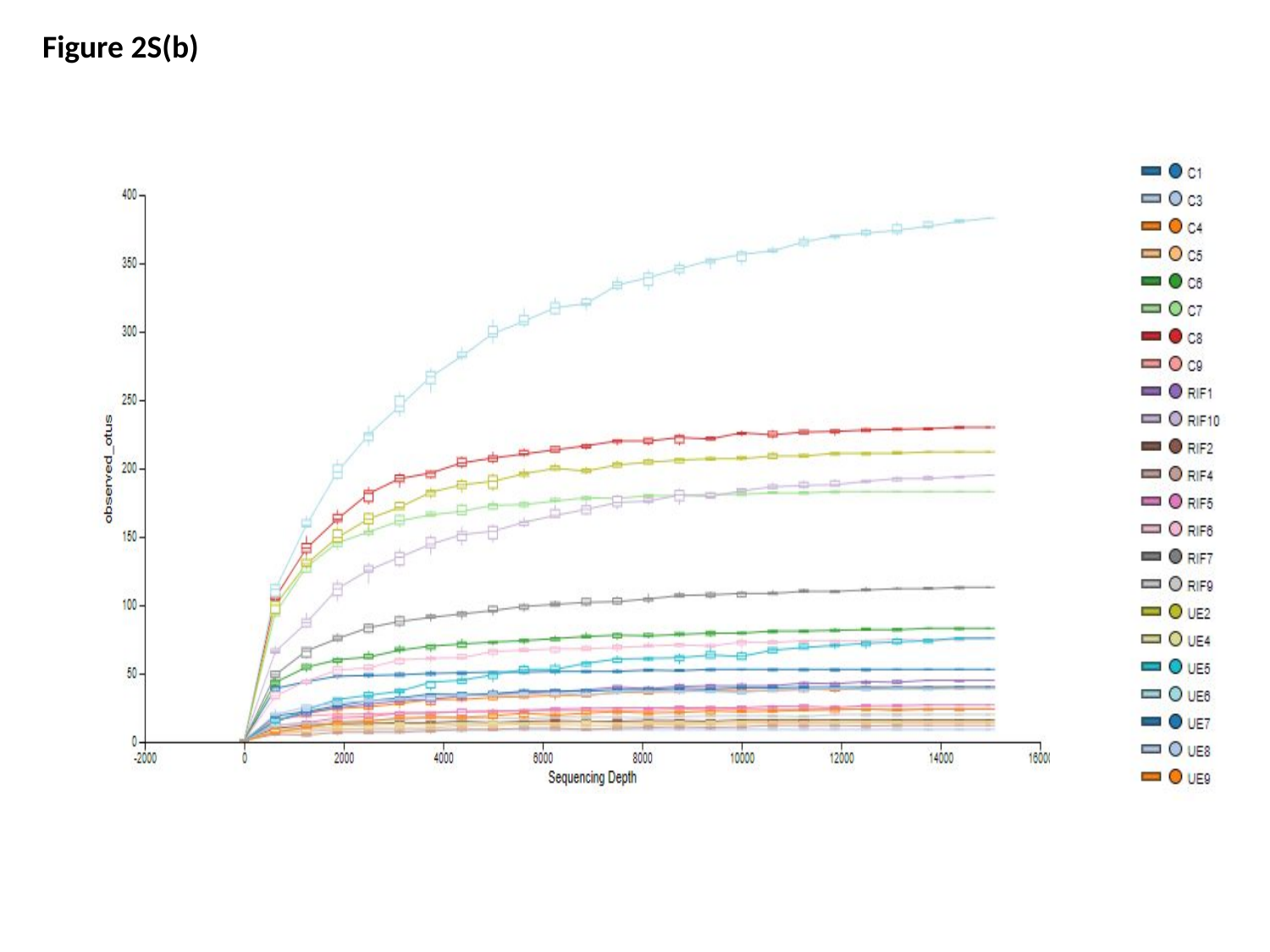

Figure 2S(b)

## Slide 6
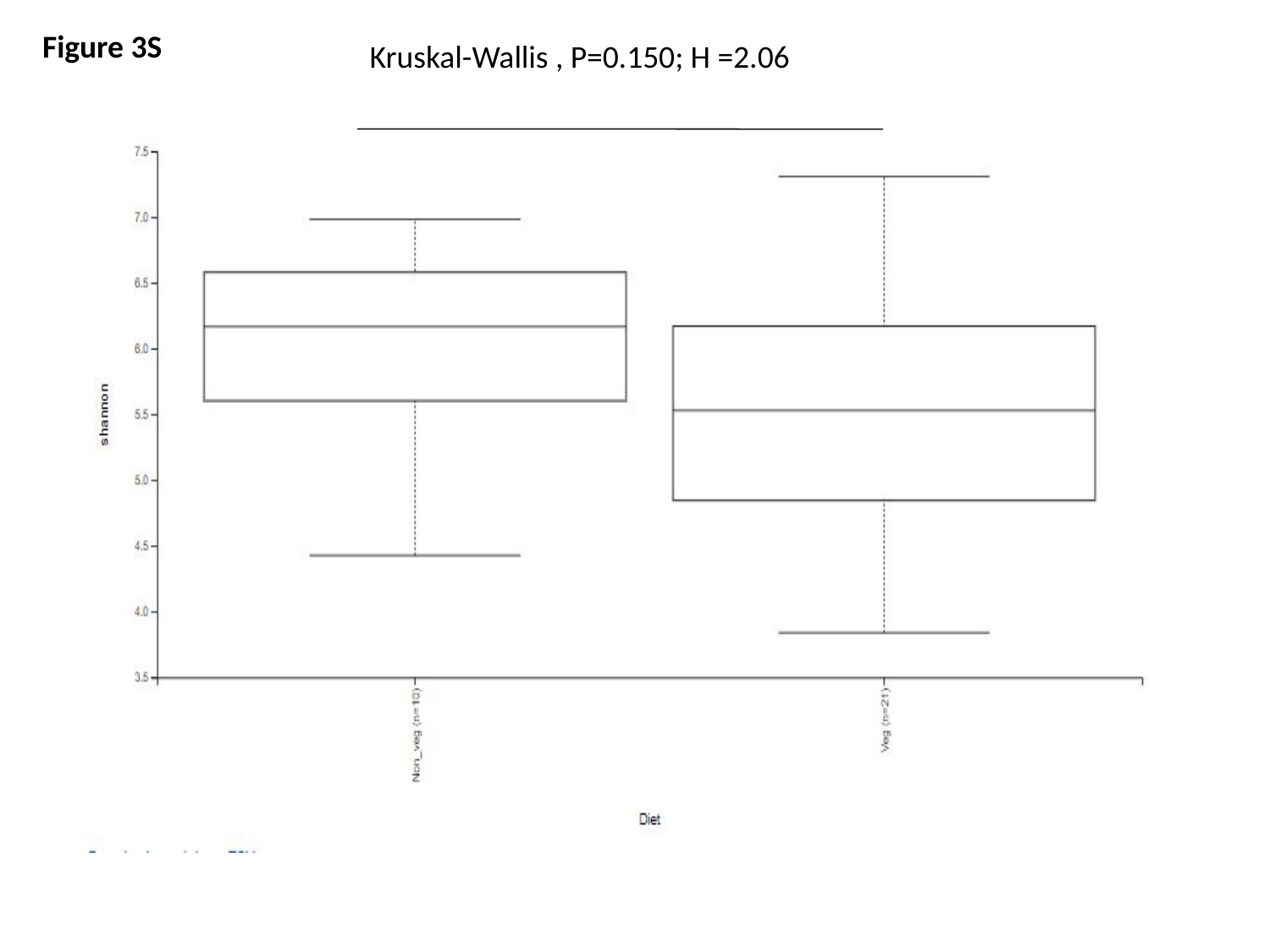

Figure 3S
Kruskal-Wallis , P=0.150; H =2.06

## Slide 7
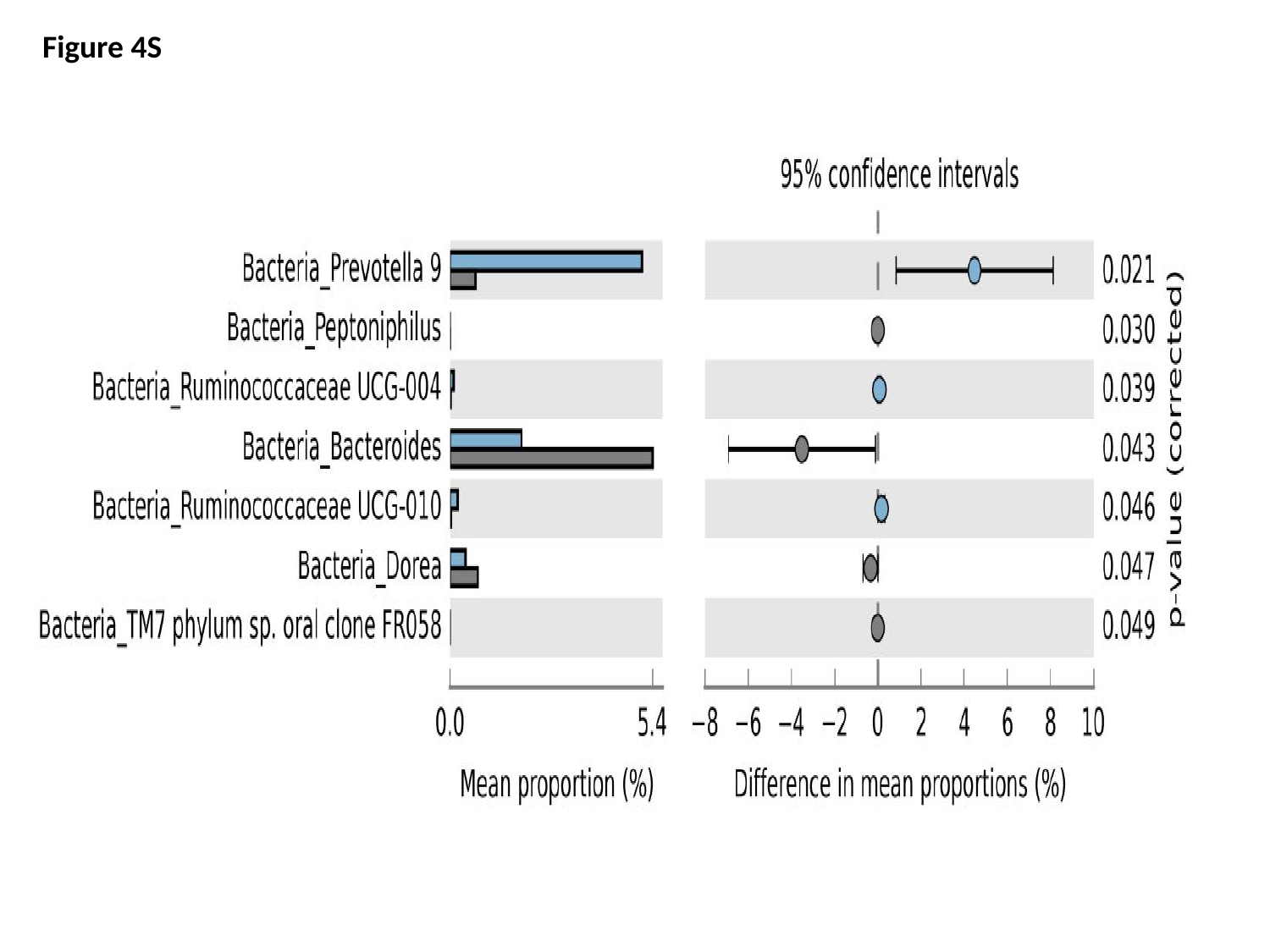

Figure 4S
